# Respiratory syncytial virus – from discovery to vaccines

**DOI:** 10.1099/jmm.0.002134

**Published:** 2026-03-23

**Authors:** Eduardo L. López, Fausto Martín Ferolla, Patricio L. Acosta

**Affiliations:** 1 Hospital de Niños Ricardo Gutiérrez, Department of Medicine, Pediatric Infectious Diseases Program, University of Buenos Aires, Buenos Aires, Argentina; 2 Consejo Nacional de Investigaciones Científicas y Técnicas, Buenos Aires, Argentina

**Keywords:** bronchiolitis, pneumonia, respiratory syncytial virus (RSV)

## Abstract

Respiratory syncytial virus (RSV) is a leading cause of hospitalization among infants and young children in the USA and worldwide. As the primary etiological agent of bronchiolitis and pneumonia in infants, RSV is the second leading cause of infant mortality after the neonatal period, causing over 200,000 deaths annually. Despite its significant impact, and although several antiviral candidates are under development, no specific therapy is currently available, and treatment remains largely supportive. While premature infants and those with chronic lung disease, congenital heart defects, Down syndrome or neuromuscular disorders are at increased risk for severe disease, the majority of hospitalizations occur in previously healthy, term-born infants. More than 97 % of childhood deaths due to RSV occur in low- and middle-income countries. RSV infections are also responsible for a substantial outpatient healthcare burden and might lead to long-term complications such as recurrent wheeze or asthma. Notably, RSV also imposes a considerable disease burden on older adults and immunocompromised individuals. The recent approval of recombinant bivalent RSV vaccines for high-risk adults and pregnant women, along with an extended half-life monoclonal antibody designed to prevent severe infection during the first 6 months of life, represents a major advancement in prevention strategies. This review explores the history, viral features and host response and clinical presentation and summarizes recent developments in RSV prevention.

## Graphical Abstract

Respiratory syncytial virus (RSV) is a leading respiratory cause of hospitalization. Symptoms are usually similar to those of the common cold, but in susceptible patients, RSV can cause severe disease and even death. The wide variation in response to RSV infection suggests that susceptibility and disease are influenced by multiple factors.

## Historical perspective

Respiratory syncytial virus (RSV) was identified in 1956 by Morris *et al*. after they isolated the virus from chimpanzees with respiratory symptoms. Initially named ‘chimpanzee coryza agent’, it was later identified in humans by Chanock *et al*. and renamed RSV because of its ability to form syncytia, which are multinuclear cells that form when cells fuse together due to the viral infection [1]. This virus rapidly became recognized as a leading cause of bronchiolitis and pneumonia in children. RSV has also been associated with long-term outcomes, including the development of recurrent wheeze and asthma [2].

Early attempts at vaccine development included the formalin-inactivated RSV vaccine, which resulted in an enhanced respiratory disease after natural RSV infection, leading to increased hospitalization rates. Moreover, two immunized infants died. This failure profoundly shaped vaccine development for decades [3].

A major milestone occurred in 1998 with the approval of palivizumab, the first monoclonal antibody against RSV, providing immunoprophylaxis for high-risk infants. However, it was the elucidation of structural details of the F-protein which allowed the development of recently approved preventive measures.

## Clinical presentation

The incubation period for RSV ranges from 2 to 8 days [4]. The clinical manifestations of RSV are highly variable, ranging from mild upper respiratory tract infection to potentially life-threatening acute lower respiratory tract involvement (ALRI) [5,6]. RSV primarily affects the upper respiratory tract, with symptoms similar to those of the common cold: nasal congestion, rhinorrhoea, cough, fever, sore throat and general malaise. Infection may progress to compromise the lower respiratory tract, causing bronchiolitis (in children under 2 years of age), pneumonia or exacerbation of chronic lung conditions (especially in adults), with symptoms like intense cough, tachypnoea, breath difficulty and wheezing [4]. Severe cases may present with lethargy or irritability, hypoxaemia and cyanosis and may require respiratory support [2,4, 5]. Severe paediatric RSV disease can be roughly classified by clinical syndrome in three age groups: neonates can present with sepsis-like illness, apnoea and/or bronchiolitis, children younger than 2 years with bronchiolitis and older children with wheezing and pneumonia. Infants can develop respiratory failure, which can be life-threatening [7]. The diagnosis of bronchiolitis could be made on the basis of history and physical examination, and radiographic or laboratory studies should not be obtained routinely [8] . After 3–6 days of upper respiratory tract clinical signs, patients can develop increased breathing effort accompanied by retractions, wheezing and tachypnoea. Infants with severe clinical progression can have feeding difficulties, hypoxaemia, apnoea, lethargy and/or irritability, indicating the need for hospitalization [7].

Co-infections with other respiratory pathogens are common during RSV infection. Bacterial co-infections (e.g. *Haemophilus influenzae*, *Streptococcus pneumoniae* and *Moraxella catarrhalis*) [9] and viral co-detections (e.g. rhinovirus and metapneumovirus) [10] are detected frequently and may contribute to increased severity. Their clinical impact remains variable across studies, but they can complicate clinical outcomes and patient management (prolonged hospitalization, antibiotic use).

## Microbial characteristics of phenotypic/genotypic features

RSV belongs to the family *Pneumoviridae*, genus *Orthopneumovirus*, and is divided into two antigenic subgroups, RSV-A and RSV-B [11].

RSV is an enveloped, negative-sense, single-stranded RNA virus with a genome of ~15,200 nucleotides. The genome has 10 genes that encode 11 proteins. The genome includes the surface glycoproteins F (fusion) and G (attachment), crucial for viral entry and immune evasion [12].

- F protein is a fusion protein that mediates virus penetration and fusion between cells to form the syncytia. The F protein has two conformations, the pre-fusion and the post-fusion forms. The pre-fusion F protein is immunogenic and a target for neutralizing antibodies [13].
- G protein mediates viral attachment to the target cells. The G protein shows variability between RSV-A and RSV-B, complicating vaccine development [14].

Additional proteins that play a key role in immune evasion include NS1 (non-structural protein 1) and NS2 (non-structural protein 2), which are associated with the suppression of type I IFN response. The N (nucleoprotein) interferes with immune activation.

The viral envelope has important implications for environmental stability: RSV is susceptible to heat and detergents, even though it can survive on surfaces for several hours, particularly in moist secretions, contributing to transmission. Importantly, RSV is inactivated by common disinfectants, including alcohol and bleach.

## Clinical diagnosis, laboratory confirmation and safety

Diagnosis of RSV is usually based on clinical presentation, particularly during the disease season (most frequently after mid-autumn and during winter). Laboratory confirmation becomes useful given that results can influence management (e.g. prompt hospitalization), especially in high-risk populations (e.g. immunocompromised patients and older adults) and infection control. The diagnosis of RSV infection requires contact isolation in hospitalized patients. Molecular testing, particularly real-time PCR, remains the gold standard due to its high sensitivity and specificity. Multiplex respiratory panels allow simultaneous detection of RSV and other common respiratory viruses, helping in the identification of co-infections. Antigen-based assays and viral cultures are also used, though with lower sensitivity [15]. Furthermore, several point-of-care molecular platforms have been developed, offering quick and accurate bedside detection and improving decision-making in emergency settings.

Even though multiple platforms for accurate detection of RSV are available, in resource-limited settings, diagnostic approaches often rely on clinical case definitions. Rapid antigen tests are frequently used despite their lower sensitivity, while centralized PCR testing may be limited by cost and infrastructure.

RSV is classified as a medium-risk biological virus and falls within biosafety level 2 (BSL2) parameters. BSL2 laboratory protocols recommend that all sample manipulations be performed in a microbiological safety cabinet. Laboratory staff handling the samples must use personal protective equipment, which includes a lab coat, closed shoes, gloves and eye protection.

## Treatment, resistance and evasion

To date, there is no evidence-based therapy for RSV infection, which remains mainly supportive: fluid hydration and nutritional and respiratory support are the foundation of evidence-based in-hospital management of RSV bronchiolitis and pneumonia [7,8, 16]. Physiological studies suggest that nebulized hypertonic saline increases mucociliary clearance and could be administered in infants hospitalized for bronchiolitis [8]. Some evidence suggests that 3% saline is safe and effective at improving symptoms of mild-to-moderate bronchiolitis after 24 h of use and reducing hospital length of stay in settings in which the duration of stay typically exceeds 3 days [8]. However, this is contradictory and results may not be clinically relevant [7,16, 17]. The evidence is clear about the ineffectiveness of bronchodilators, epinephrine and corticosteroids for the treatment of bronchiolitis [7,8, 16,18]. Moreover, the use of corticosteroids could worsen the course of the infection and may prolong viral shedding. Antiviral therapy has not shown much promise in the treatment of RSV infection, with the dilemma that the delay in treatment is too long for antivirals to effectively interrupt viral replication and therefore prevent an associated immune response [7,18]. Ribavirin is a synthetic nucleoside analogue that interferes with viral replication through inhibition of viral RNA-dependent RNA polymerase, induction of lethal mutagenesis and impaired nucleotide synthesis, leading to reduced RSV viral replication. Despite this potential antiviral effect, ribavirin remains restricted to severe RSV infection in highly vulnerable immunocompromised populations [19]. Regarding safety, ribavirin is associated with haemolytic anaemia, which can be clinically significant, especially with systemic administration; other adverse effects include teratogenicity, hepatotoxicity, electrolyte disturbances, rash and gastrointestinal symptoms. Aerosolized administration can cause respiratory irritation, bronchospasm and headache and poses potential occupational exposure risks to healthcare workers.

Respiratory support is the milestone of life-saving treatment for severe RSV. One of the pillars of treatment in these patients is oxygen therapy, both with low-flow and high-flow systems. High-flow nasal cannula (HFNC) oxygen therapy has been increasingly used in the past decade for management [7,20]. An HFNC is an open system that delivers HF oxygen with optimal temperature and relative humidity. Its main action mechanisms include flushing the nasopharyngeal dead space, delivering flow according to the increased inspiratory demand, delivering a measurable fraction of inspired oxygen (FiO_2_) and generating continuous positive airway pressure. Observational and experimental studies reported a reduction in the admission rate to the paediatric intensive care and mechanical ventilation requirement associated with its use. Therefore, in addition to a better tolerance and a lower cost compared to non-invasive mechanical ventilation, support with HFNC appears increasingly more interesting [20]. However, its use must be properly protocolized and should not delay the timely admission of patients to intensive care and mechanical respiratory support [7].

RSV’s genome is susceptible to mutations, which can affect the binding of monoclonal antibodies and lead to resistance against antiviral agents. A study has shown that mutations in the fusion (F) protein may reduce the efficacy of MEDI8897, an anti-RSV fusion antibody [21]. Another study found that some RSV variants showed resistance during the drug-resistance evaluation of presatovir, an RSV fusion inhibitor [22]. Moreover, Fouratti *et al*. reported recently rare RSV-B F-protein substitutions associated with resistance to nirsevimab. However, these findings were uncommon and have not been linked to sustained transmission or diminished clinical effectiveness. Overall, while resistance is biologically plausible, clinically significant resistance remains rare [23]. To ensure detection of emerging resistance, ongoing surveillance efforts must incorporate routine RSV sequencing, especially in infants receiving nirsevimab and immunocompromised populations where selection pressure may be higher.

Host innate immune responses against RSV can be activated through several pattern recognition receptors (PRRs) [24]. Activation of PRRs usually leads to IFN type I (IFN-I) secretion, but RSV in particular is a poor stimulator of IFN-I^24^. Two proteins encoded by RSV (NS1 and NS2) suppress IFN-I production, impairing early antiviral signalling. Furthermore, other proteins such as G protein of RSV have been demonstrated to modulate cytokine and chemokine expression and immune cell function. RSV N protein encapsidates viral RNA and also has been shown to interfere with immune signalling during immune system activation [24,25].

## Pathogenic strategies (host range, host response, transmission, infection and virulence factors)

The primary host for RSV is humans. RSV can infect non-human primates and rodents under experimental conditions [1].

RSV targets respiratory epithelial cells and is transmitted primarily through droplets and direct contact. Upon entry, the G and F proteins facilitate attachment and fusion, leading to syncytia formation, which enables cell-to-cell spread while avoiding immune defenses [1].

The host’s innate immune response is initiated by Toll-like receptors (TLR3 and TLR4) that recognize viral components. This leads to the release of pro-inflammatory cytokines (IL-6, IL-8 and TNF-*α*) and IFNs to limit viral replication. However, RSV actively modulates the immune response through NS proteins that inhibit IFN production, crucial for early viral control [24].

Adaptive immunity involves both humoral and cellular responses. CD4 and CD8 T-cells are essential for resolving the RSV infection, while antibodies protect against future infections. CD8 T-cells play a central role by secreting cytokines and killing infected cells. Studies have shown that a proper T-cell response is essential for viral clearance, with CD8 T-cells required for complete viral elimination [26]. Additionally, other immune cells, such as Th1 and Th2 CD4 T-cells, and the balance of cytokine responses, may influence disease severity, with Th2 responses contributing to increased mucus production and airway hyperresponsiveness [1,27, 28].

RSV induces only partial and transient immunity, failing to generate long-lasting protection. Antibody levels decline over time, allowing for frequent reinfections. The immaturity of the immune system in young children and immunosenescence in older adults may also contribute to this vulnerability. Furthermore, RSV primarily infects the mucosal surfaces of the respiratory tract, where immune responses – particularly mucosal IgA – are generally less durable than systemic immunity, resulting in reduced antibody persistence. Lastly, although memory B-cells are generated, the magnitude and longevity of immune memory specific to RSV remain limited, which contributes to the short duration of protective antibody responses. Pre-F is the active conformation of the F protein, and antibodies against pre-F have demonstrated high neutralizing activity. Neutralizing antibodies may reduce viral replication at an earlier stage of disease, diminishing the risk of hospitalization; however, they may have a limited effect on disease severity once the virus reaches the lungs [1,29].

## Epidemiology, prevention and risk groups

RSV causes seasonal outbreaks worldwide, from May to September in the southern hemisphere and from October to May in the northern hemisphere. However, following the severe acute respiratory syndrome coronavirus 2 pandemic, several countries reported atypical RSV circulation, including delayed peaks and expanded season lengths [30].

RSV is a major cause of ALRI, with an estimated 33 million cases annually in children under five, resulting in over 3 million hospitalizations and more than 200,000 deaths around the world [31]. Adults over 65 years old also represent a vulnerable population, with an estimated 250,000 hospitalizations and 14,000 deaths annually due to RSV-related complications [32]. Although RSV affects populations globally, the burden of severe disease and mortality is higher in low- and middle-income countries, where over 97 % of RSV-related deaths occur [31]. Several structural and systemic barriers contribute to this disparity, including limited access to the healthcare system and underdiagnosis due to the lack of molecular tests or intensive care units in resource-limited settings [2]. Moreover, social determinants such as lacking a sewage system, exposure to indoor smoke or malnutrition further exacerbate RSV vulnerability in these regions [2,10].

Preventive measures include monoclonal antibodies and vaccination strategies. Palivizumab, the first monoclonal antibody approved for RSV, targets the F protein and is administered to high-risk infants, such as those born prematurely or with chronic lung or congenital heart disease. However, its requirement for monthly dosing has limited its widespread use [33]. Nirsevimab, a next-generation monoclonal antibody, has demonstrated 100-fold greater neutralizing avidity than palivizumab and has a longer-lasting effect, up to 6 months. A Spanish study found that nirsevimab was 82% effective in preventing hospitalizations and 86.9% effective against severe RSV-related ALRI [34].

RSV vaccine development has faced several challenges [3], but in recent years, substantial advancements have been made. To date, three vaccines have been approved.

Arexvy (GlaxoSmithKline) was approved by the US Food and Drug Administration (FDA) in May 2023 for the prevention of ALRI caused by RSV in adults aged 60 and older, as well as in adults aged 50–59 who are at increased risk [29]. Moreover, in August 2023, the FDA also approved Abrysvo (Pfizer), making it the first vaccine authorized for use in pregnant women [29]. Maternal vaccination offers a preventive strategy by conferring passive immunity to infants through transplacental antibody transfer. In March 2024, Argentina became the first country to implement a national programme for the immunization of pregnant individuals between 32 and 36 weeks of gestation as the primary strategy to prevent RSV disease among infants from birth through 6 months of age [35]. This vaccine is also approved by the FDA and the European Medicines Agency (EMA) for the immunization of individuals aged 18–59 years who are at increased risk for RSV-related ALRI, as well as for those aged 60 years and older [36]. A third vaccine, mRESVIA (Moderna), an mRNA-based vaccine, has recently been approved for use in older adults [37].

However, the implementation of these novel preventive strategies faces several challenges, including cost, cold-chain requirements, disparities in access and increasing vaccine hesitancy. Low- and middle-income countries face additional barriers, such as limited maternal care coverage, reduced diagnostic capacity and logistical constraints.

A summary of the preventive measures currently approved is presented in Table 1.

**Table 1.** Comparison of prevention strategies

Strategy	Type	Target population	Schedule	Duration of protection	Advantage	Limitation
**Palivizumab (Synagis**)	Monoclonal antibody	High-risk infants	Monthly IM dose	30 days	Reduction of hospitalizations in high-risk infants	High cost; monthly dosing; limited to high-risk groups
**Nirsevimab (Beyfortus**)	Monoclonal antibody (extended half-life)	Infants entering first RSV season; high-risk older infants	Single IM dose (before RSV season)	~6 months	Single dose High effectiveness against hospitalization	Supply/distribution limitations Cost and access challenges
**Maternal vaccination (Abrysvo**)	Maternal immunization; passive protection for infants	Pregnant woman	Single IM dose at 26^*^/32^†^–36 weeks gestation	Passive protection for infants up to 6 months	Protects infants via transplacental antibodies	May not protect preterm infants if delivered before antibody transfer
**Adult vaccination (Arexvy, Abrysvo for adults, mRESVIA**)	Active immunization (protein or mRNA vaccines)	Older adults (≥60) and adults 18–59 at increased risk	Single IM dose (seasonal/one-off per guidance)	Evidence of protection at least through one season	Reduces RSV hospitalizations in older adults	Durability and re-vaccination schedules under study; cost and programme implementation

*EMA.

†FDA.

### Risk groups

Several groups have been described to have an increased risk for RSV severe disease. Among them are age (<12 months or >65 years old), sex (male), prematurity, low birth weight, lack of breastfeeding, underlying chronic illness (heart, lung, neurological or immune disease and Down’s syndrome), genetic background and nutritional or environmental variables (vitamin D deficiency, maternal alcohol consumption during pregnancy, exposure to tobacco, indoor air pollution, crowding conditions and diets rich in carbohydrates) [10,27, 38,43].

## Open questions

1. What host genetic, immunological or environmental factors predispose individuals to severe RSV outcomes?
2. How do early-life RSV infections influence long-term airway remodelling and the development of recurrent wheezing or asthma?
3. Does RSV coinfection with other respiratory pathogens influence disease severity and treatment outcomes?
4. Which antiviral candidates currently in development have the highest likelihood of becoming a broadly effective and safe treatment?
5. What are the most effective approaches to reduce vaccine hesitancy among pregnant individuals and caregivers?

## Abbreviations

ALRI: acute lower respiratory tract involvement
BSL2: biosafety level 2
EMA: European Medicines Agency
FDA: Food and Drug Administration
HFNC: high-flow nasal cannula
IFN-I: IFN type I
PRRs: pattern recognition receptors
RSV: respiratory syncytial virus
